# Biomarkers for Early Detection of Cancer: Molecular Aspects

**DOI:** 10.3390/ijms24065272

**Published:** 2023-03-09

**Authors:** Paramjit S. Tappia, Bram Ramjiawan

**Affiliations:** 1 Asper Clinical Research Institute and Albrechtsen Research Centre, St. Boniface Hospital, Winnipeg, MB R2H 2A6, Canada; bramjiawan@sbrc.ca; 2 Department of Pharmacology and Therapeutics, Rady Faculty of Health Sciences, Max Rady College of Medicine, University of Manitoba, Winnipeg, MB R3E 0T6, Canada

According to the World Health Organization, cancer is a leading cause of death worldwide, representing almost 10 million deaths in 2020. In other words, approximately one death out of every six can be attributed to cancer. Furthermore, around 30% of deaths from cancer are linked to tobacco use, high body mass index, excessive alcohol consumption, a diet containing low fruit and vegetable intake, and physical inactivity [1]. Infections due to human papillomavirus (HPV) and hepatitis have been linked to one-third of cancer cases in low- and lower-middle-income countries [2]. The most common types of cancer are breast, lung, colon, rectum, and prostate cancers.

Although the American Cancer Society recommends that those over 40 years of age undergo annual cancer check-ups, this recommendation is usually not heeded and thus, as a consequence, when patients show cancer symptoms at a later stage of development, their survival tends to be poor [3]. Indeed, many cancers can be cured if detected early and treated appropriately and effectively. The current standards for determining and verifying the presence of cancer involve computed tomography, magnetic resonance imaging, and ultrasound technology. These techniques are expensive, often inaccessible, involve exposure to ionizing radiation, and have an unacceptable level of false positives. 

Recently, a number of blood-based cancer assays that detect protein, microRNA, circulating DNA, and methylated DNA biomarkers have been developed. Unfortunately, most are specific to late-stage cancer. In addition, biopsies for molecular and biomarker diagnosis are invasive and uncomfortable for patients. Furthermore, the issue of overdiagnosis is a limiting factor, causing anxiety for patients and further increasing expense for the healthcare system. Thus, researchers have turned their attention to developing low-cost, high-throughput, early detection tools with high selectivity and specificity for cancer detection. This Special Issue provides insight and discusses recent advances in molecular markers for the early detection of different types of cancer. Novel approaches in the early detection of different cancer types will also be described in terms of their potential for the screening and surveillance of cancer to improve prognostic value, survival, and quality of life.

In this Special Issue, nine state-of-the-art and outstanding papers are presented: four original research manuscripts and five literature reviews which provide insights into molecular diagnostics in cancer detection as well as the use of molecular markers for the assessment of tumor response to cancer treatment. In fact, despite the advances in chemotherapy, the persistent issue of the resistance and non-responsiveness of tumors to treatment regimens is still of major concern [4]. Indeed, the assessment of tumor response to therapy is of critical importance as it permits a prospective end-point evaluation and provides a guide for clinicians to make future treatment decisions. Furthermore, current practices in the early evaluation of chemotherapy are insufficient [5].

In this regard, in the paper by Koguchi et al. [6] the predictive value of phosphoglycerate kinase 1 (PGK1) was investigated for treatment outcomes in first- and second-line chemotherapy in patients with advanced urothelial cancer. It was observed that the non-response rates to first-line chemotherapy were significantly higher in patients with a high expression of PGK1 in the nucleus than in those with a low expression. In addition, this study demonstrated the clinical utility of the higher nuclear PGK1 gene expression as predictive of resistance to platinum-based chemotherapy in patients with advanced urothelial cancer. This is an interesting study as the expression levels of a single nuclear gene may have clinical value in assessing tumor response to chemotherapy.

The issue of tumor resistance to drugs is further highlighted in a study by Gujrati et al. [7], which was conducted in African American men with prostate cancer. The nuclear mammalian target of rapamycin (mTOR) is known to regulate cell proliferation, protein synthesis, metabolism, apoptosis, and autophagy [8], and was shown to be elevated in patients with prostate cancer as well as other advanced cancers (lung, breast, and colon). The dysregulation of microRNA, specifically microRNA-99b-5p, is known to be linked with prostate cancer [9], and in the study by Gujrati et al. [7] it was found to be downregulated. These investigators went on to demonstrate, in vitro, that transfection with microRNA-99b-5p significantly reduced the expression levels of nuclear mTOR and androgen receptor (AR). In addition, the overexpression of miR-99b-5p targets/inhibits the AR-mTOR axis, inducing cell apoptosis. It was thus concluded that reciprocal miR-99b-5p/nuclear mTOR pairing may be a more precise diagnostic/prognostic biomarker for aggressive prostate cancer and that the AR-mTOR axis may serve as a novel therapeutic strategy to induce apoptosis and overcome chemoresistance in aggressive prostate cancer. This is an important study that affirms the importance of AR-mTOR signal transduction at the nuclear level that could be a key target for tumor regression during chemoresistance in the aggressive form of prostate cancer.

On the other hand, mutations in DNA damage response genes (DDRG) can predict the response to cisplatin-based chemotherapy. Herrmann et al. [10] examined the value of RNA expression levels of several key DDRGs (*BCL2*, *BRCA1*, *BRCA2*, *ERCC2*, *ERCC6*, *FOXM1*, *RAD50*, *RAD51*, and *RAD52*) in patients with muscle-invasive bladder cancer and validated these with The Cancer Genome Atlas (TCGA). It was determined that the low expression of *RAD52* correlated with decreased disease-free survival in patients, which was validated by TCGA. This observation was more apparent in the subset of patients who received adjuvant cisplatin-based chemotherapy and thus these investigators suggested that RAD52 gene expression could be a promising marker for response to adjuvant cisplatin-based chemotherapy.

Globally, colorectal cancer (CRC) is the third most common cancer. A panel of biomarkers may offer more specificity and selectivity than single gene targets for the early detection of this type of cancer. In this regard, a systematic survey of gene signatures that may have clinical utility as prognostic and early detection biomarkers in CRC has been explored. In the study by Ghatak et al. [11], four genes (*BDNF, PTGS2, GSK3B*, and *CTNNB1*) were found to be upregulated and one gene (*HPGD*) was found to be downregulated in primary tumor tissues. An important feature of this article was the observation that the five-gene signature was linked to poor overall survival. Furthermore, with the inclusion of the TNM stage and sex, a greater predictive value was obtained, thus providing a powerful tool for personalized risk assessment in CRC patients. This study highlighted the value of a biomarker panel with a higher predictive capability, thus improving prognosis.

Studies have shown that COVID-19 is associated with the amplified inflammatory response, leading to a pro-inflammatory phenotype that has been linked to several common age-related diseases, including cancer [12]. Interestingly, it has also been suggested that COVID-19 escalates the aging process [13]. The paper by Tyagi et al. [14] proposes that COVID-19 mimics the pulmonary dysfunction in Duchenne muscular dystrophy (DMD). The common feature of both of these conditions is the involvement of neopterin (NPT), which has been suggested as a prognostic marker in human malignancies [15]. In addition, the DMD gene has been tentatively implicated in the development of all major cancer types [16,17]. The probable mechanisms leading to respiratory abnormalities have been reviewed. Studies with DMD and genetically engineered humanized mice that were administered with the spike protein (SP) of SARS-CoV-2 revealed an increase in the levels of NPT, TNF-α, HDAC, IL-1β, CD147, and MMP9 in the lung tissue of the animals, subsequently accompanied by fibrosis of the diaphragm depicting a decreased oscillation phenotype. Such factors could be considered important diagnostic, prognostic, and/or predictive markers for disease, but the central factor appears to be NPT. It would be interesting to investigate the relationship between COVID-19, NPT, and the development of lung cancer.

In the U.S., ovarian cancer ranks fifth in cancer deaths among women. It is very unfortunate that ovarian cancer frequently presents with a lack of specific symptoms and there is a paucity of effective/reliable biomarkers for the early detection of this cancer type. Indeed, cancer antigen 125 (CA125) is widely used, but it is highly unspecific. This has led to the development of novel biomarker panels with improved sensitivity and specificity for ovarian cancer detection. A review of existing and recent advancements in the development of biomarkers for the early detection of ovarian cancer are highlighted in the article by Zhang et al. [18]. These continuing investigations aim to establish novel molecular biomarkers, such as autoantibodies, ctDNAs, miRNAs, and DNA methylation signatures, to facilitate the earlier detection of ovarian cancer and improve prognosis.

Bladder cancer is the fourth most common cancer in men. While current screening methods for primary bladder cancer involving cystoscopy and urine cytology are generally accepted, their diagnostic accuracy along with the high rates of false negatives are unsatisfactory. Subsequently, the diagnostic/predictive utility of liquid biopsy has gained interest to increase specificity, sensitivity, and selectivity for the early detection of primary bladder cancer, Accordingly, Koguchi et al. [19] reviewed the literature on the diagnostic power of liquid biopsy for the early detection of this type of cancer, with particular focus on circulating tumor cells, urinary cell-free DNA, and urinary microRNA. Although no definitive conclusions could be drawn, the value of a panel consisting of microRNA and cell-free DNA genes in the urine was encouraging; however, to be implemented as a screening/surveillance tool for the early detection of primary bladder cancer, validation is required with a much greater sample size. Even though both new bladder cancers and deaths from bladder cancer have been declining [20], the establishment of a non-invasive, cost-effective, reliable, and accurate test for the early detection of primary bladder cancer will significantly contribute to a further reduction and improved prognosis for this type of cancer. Lung cancer is the second most common cancer in both men and women in the U.S. However, there is still a lack of optimal early detection methods for lung cancer; as a result, most new lung cancer cases are diagnosed at advanced or late stages and the five-year survival rate is alarmingly low. Haince et al. [21] proposed a paradigm shift to metabolomics from the molecular- and proteomic-based markers used for screening lung cancer. While the current early detection methods such as chest X-rays followed by bronchoscopy, sputum analysis followed by cytological analysis, and low-dose computed tomography have some limitations, the authors discuss the feasibility of metabolomic fingerprinting as an accessible, low-cost, and high-throughput blood-based screening test in populations at risk for lung cancer. Nooreldeen and Bach [22] elaborated further on screening high-risk populations, including smokers and people exposed to toxic fumes either occupationally or through the environment. In their review, the authors discuss the pro and cons of existing methods currently used in clinical practice in the diagnosis of lung cancer and critically analyze the utility of testing body fluids for the presence of biomarkers that could be predictive for the development and progression of cancer.

Both the early detection and an ability to assess the tumor response to treatment are critical aspects in the field of cancer. This Special Issue includes contributions from global experts and has focused on urothelial, colorectal, bladder, ovarian, and lung cancers. Although not discussed in the current Special Issue, it should be mentioned that breast cancer, which is the most common diagnosed cancer globally [23], is a highly heterogeneous disease and even though advances have been made in the early detection and understanding of its molecular bases, around a third of cases with early stage breast cancer have metastatic disease [24]. With evolving molecular techniques and increased understanding of the different subtypes of breast cancer (based on genetic as well as epigenetic markers), establishing predictive biomarkers and having the capacity to assess breast tumor response to treatment is also of critical importance.

Taking the contributions to this Special Issue into consideration, it is clear that the discovery and validation of novel, highly specific and selective biomarkers for the early detection of different types of cancer is urgently required. Furthermore, the development of tests that are effective in determining the efficacy of therapeutic interventions is also warranted, as an improved assessment of the response to treatment will allow for improved management and lower risk of toxicity for patients undergoing cancer treatment. It is hoped that experts within the field of cancer diagnostics and those that have a general interest in biomarkers and human health will find the information presented in this Special Issue useful, educational, and insightful, and that it will prompt further exploration and advancement in the understanding of the clinical value of biomarkers as predictive and diagnostic tools and validate and establish specific biomarkers for routine use and application in clinical oncology practice.
